# A Systematic Review and Meta-analysis of Childhood Leukemia and Parental Occupational Pesticide Exposure

**DOI:** 10.1289/ehp.0900582

**Published:** 2009-05-19

**Authors:** Donald T. Wigle, Michelle C. Turner, Daniel Krewski

**Affiliations:** McLaughlin Centre for Population Health Risk Assessment, University of Ottawa, Ottawa, Ontario, Canada

**Keywords:** child, leukemia, meta-analysis, occupational exposure, pesticides

## Abstract

**Objectives:**

We conducted a systematic review and meta-analysis of childhood leukemia and parental occupational pesticide exposure.

**Data sources:**

Searches of MEDLINE (1950–2009) and other electronic databases yielded 31 included studies.

**Data extraction:**

Two authors independently abstracted data and assessed the quality of each study.

**Data synthesis:**

Random effects models were used to obtain summary odds ratios (ORs) and 95% confidence intervals (CIs). There was no overall association between childhood leukemia and any paternal occupational pesticide exposure (OR = 1.09; 95% CI, 0.88–1.34); there were slightly elevated risks in subgroups of studies with low total-quality scores (OR = 1.39; 95% CI, 0.99–1.95), ill-defined exposure time windows (OR = 1.36; 95% CI, 1.00–1.85), and exposure information collected after offspring leukemia diagnosis (OR = 1.34; 95% CI, 1.05–1.70). Childhood leukemia was associated with prenatal maternal occupational pesticide exposure (OR = 2.09; 95% CI, 1.51–2.88); this association was slightly stronger for studies with high exposure-measurement-quality scores (OR = 2.45; 95% CI, 1.68–3.58), higher confounder control scores (OR = 2.38; 95% CI, 1.56–3.62), and farm-related exposures (OR = 2.44; 95% CI, 1.53–3.89). Childhood leukemia risk was also elevated for prenatal maternal occupational exposure to insecticides (OR = 2.72; 95% CI, 1.47–5.04) and herbicides (OR = 3.62; 95% CI, 1.28–10.3).

**Conclusions:**

Childhood leukemia was associated with prenatal maternal occupational pesticide exposure in analyses of all studies combined and in several subgroups. Associations with paternal occupational pesticide exposure were weaker and less consistent. Research needs include improved pesticide exposure indices, continued follow-up of existing cohorts, genetic susceptibility assessment, and basic research on childhood leukemia initiation and progression.

Although leukemia is the most common childhood cancer, the only established modifiable risk factor is prenatal or childhood exposure to ionizing radiation (Belson et al. 2007; Doll and Wakeford 1997). Acute lymphocytic leukemia (ALL) comprises about 80% of all childhood leukemia cases, the remainder being mainly acute myeloid leukemia (AML) (Borugian et al. 2005). All known risk factors, including ionizing radiation, sex, race, Down syndrome, and other genetic syndromes, account for < 10% of all childhood leukemia cases (Buffler et al. 2005). A narrative review concluded that recent epidemiologic studies are consistent with those reviewed previously (Zahm and Ward 1998) and support associations between childhood leukemia and parental pesticide exposure before and during pregnancy and childhood exposure to household insecticides (Infante-Rivard and Weichenthal 2007). In a recent meta-analysis, childhood leukemia was weakly associated with preconceptual and overall paternal smoking (Lee et al. 2009). Other potential risk factors include preconceptual paternal occupational exposure to solvents (Buckley et al. 1989), motor exhaust fumes (Vianna et al. 1984), or electromagnetic fields (Feychting et al. 2000; Pearce et al. 2007); prenatal maternal alcohol consumption (for AML) (Shu et al. 1996); and reduced occurrence of common infections during childhood (Ma et al. 2005). Prenatal maternal occupational electromagnetic field exposure was linked to childhood leukemia in a Canadian case–control study (Infante-Rivard and Deadman 2003) but not in two other case–control studies (Feychting et al. 2000; Sorahan et al. 1999).

Preconceptual paternal occupational or environmental exposures have not been established as causes of any childhood cancer. Although prenatal maternal exposure to ionizing radiation can cause childhood leukemia, there is little evidence that preconceptual paternal ionizing radiation exposure is a risk factor (Draper et al. 1997; Johnson et al. 2008; McLaughlin et al. 1993; United Nations Scientific Committee on the Effects of Atomic Radiation 2000). Paternal smoking has also been linked to increased risks of childhood brain cancer and lymphomas (California Environmental Protection Agency 2005).

Occupational exposures of reproductive-age adults to pesticides may substantially exceed those from other sources. Serum hexachlorobenzene (HCB) levels among men occupationally exposed to airborne HCB in Spain were 6-fold higher than those of unexposed men (Sala et al. 1999). Geometric mean peak daily urinary pesticide levels in agricultural applicators were notably higher than those of their spouses [2,4-D (2,4-dichlorophenoxyacetic acid), 61 vs. 1 ppb; glyphosate, 3 vs. < 0.5 ppb; chlorpyrifos, 19 vs. 5 ppb] (Mandel et al. 2005). Pregnant women employed as farm fieldworkers in California had significantly higher prenatal urinary organophosphate insecticide metabolite levels compared with pregnant women in the general U.S. population; mean total dialkyl phosphate urinary metabolite levels in these two groups were 113 and 70.5 nmol/L, respectively (Bradman et al. 2005).

Although the present study focuses on preconceptual paternal and prenatal maternal exposure, children may have relatively high exposures to certain pesticides. In the 1999–2000 cycle of the National Health and Nutrition Examination Survey, children 6–11 years of age had higher levels of urinary 3,5,6-trichloro-2-pyridinol (TCPy, a chlorpyrifos metabolite) than did adolescents or adults (Barr et al. 2005). Farm children in Iowa had higher urinary atrazine levels compared with nonfarm children (0.71 vs. 0.46 μg/L, *p* < 0.001) (Curwin et al. 2007). Among inner-city children 3–6 years of age in Minneapolis, the highest measured blood levels of heptachlor epoxide, oxychlordane, 1,1-dichloro-2,2-bis(*p*-chlorophenyl)ethylene (*p*,*p*-DDE), and *trans*-nonachlor approached or exceeded the 95th percentile levels of older children and adults in national surveys (Sexton et al. 2006). In North Carolina and Ohio, preschool children had urinary pentachlorophenol (PCP) levels more than 10-fold those predicted from multimedia PCP levels in homes and daycare centers (Wilson et al. 2007).

In this systematic review and meta-analysis we synthesize currently available epidemiologic evidence on the relationships between childhood leukemia and paternal or maternal occupational pesticide exposure. A related report addresses childhood leukemia and parental or childhood residential pesticide exposure (Turner et al. 2009).

## Materials and Methods

This systematic review and meta-analysis was conducted according to a protocol designed by two of us (D.T.W. and M.C.T.).

### Literature search

The literature search and selection processes were conducted simultaneously for studies of childhood leukemia and parental occupational and parental or childhood residential pesticide exposure. The search strategy [see Supplemental Material, Appendix 1, available online (doi:10.1289/ehp.0900582.S1 via http://dx.doi.org/)] was applied to OVID MEDLINE database (1950–2009 March week 3) and OVID MEDLINE database of in process and other nonindexed citations (1950 to March 31 2009) (Ovid 2009) and then adapted to search the OVID EMBASE (1980–2009 week 13) (Ovid 2009), TOXNET (U.S. National Library of Medicine 2009), OpenSigle (2009), and ProQuest Digital Dissertations and Theses (2009). We used the following MeSH (medical subject heading) terms and key words:

- Exposure: exp Environmental Exposure/, exp Environmental Pollutants/, exp Pest Control/, exp Pesticides/, pesticid$.tw, herbicid$.tw, insecticid$.tw, fungicid$.tw
- Population: exp Child/, exp Adolescent/, exp Infant/, child$.tw, adolescen$.tw, infant?.tw, newborn?.tw, youth.tw, teenage$.tw
- Outcome: exp Hematologic Neoplasms/, exp Leukemia/, leuk?emia$.tw.

Search terms were grouped according to the Boolean operators “OR” and “AND.” We screened all titles and abstracts to determine their suitability and then applied inclusion/exclusion criteria to the complete articles and resolved discrepancies by consensus. We attempted to contact the corresponding author of reports that did not include confidence intervals (CIs) or other essential information. We also searched the reference lists of all included studies.

### Inclusion and exclusion criteria

**Figure 1:**
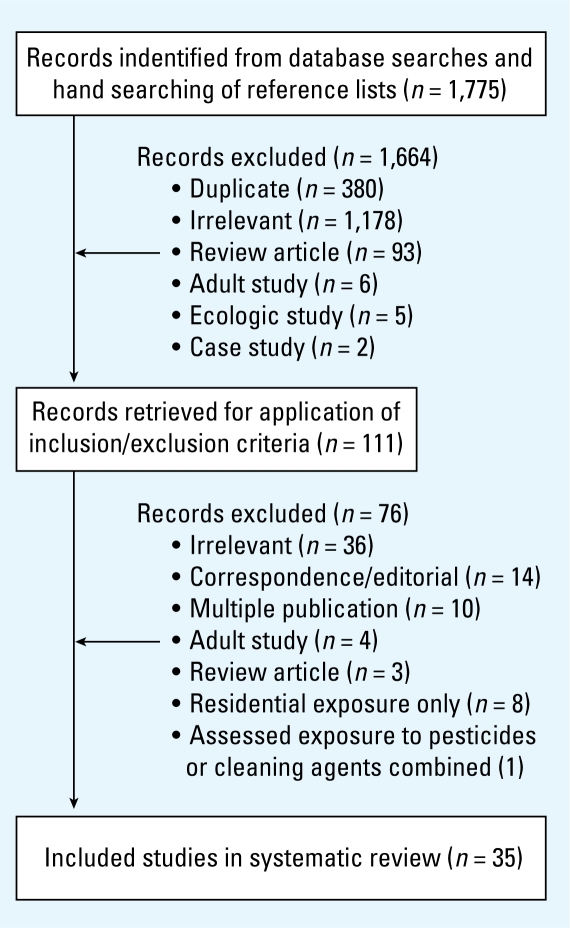
Literature search results.

Inclusion criteria were *a*) original epidemiologic research on childhood leukemia, *b*) use of an analytic design (case–control or cohort), and *c*) availability of at least one index of paternal or maternal occupational pesticide exposure. Studies that included a history of occupation in farming or other jobs with likely pesticide exposure and those with self-reported or documented information on occupational pesticide exposure were included. Reports were excluded if only ecologic data were collected and analyzed, or if more recent/relevant reports of the same study were available; case reports and cluster investigations were also excluded. No language criteria restrictions were applied. A flowchart of the selection process is provided in Figure 1.

### Data abstraction

D.T.W. and M.C.T. independently extracted key data from all included studies using a data abstraction form piloted before the present study was undertaken. Data categories comprised referencing, study design, subject selection, exposure assessment, statistical analysis, and results. For each included study, we identified a single exposure index per parent and pesticide category (unspecified, insecticides, herbicides, fungicides). In 15 of 27 included studies, paternal occupational pesticide exposure during the period up to 2 years before conception was well defined; maternal occupational pesticide exposure during pregnancy was well defined in 15 of 16 included studies [Table 1; also see Supplemental Material, Appendix 4 (doi:10.1289/ehp.0900582.S1)]. For studies reporting more than one risk estimate relevant to a given meta-analysis, a single odds ratio (OR) was selected based on *a*) specificity of the exposure index (e.g., a self-reported occupational pesticide exposure was preferred to job title alone and *b*) intensity or duration of exposure (e.g., an index based on frequency or duration of use was used instead of one based on ever vs. never exposed). Although analysis of exposure duration or intensity reduces the numbers of exposed subjects in the highest exposure category (compared with analyses of ever/never exposed), only three studies had such data, and the numbers of highly exposed case parents included 11 mothers and 27 fathers (Buckley et al. 1989), 5 fathers (Heacock et al. 2000), and 2 mothers (Steinbuch 1994) [for key characteristics of these and other included studies, see Supplemental Material, Appendix 2 (doi:10.1289/ehp.0900582.S1)].

### Quality assessment

We modified the assessment tool of Downs and Black (1998), a checklist for assessing the methodological quality of health care interventions, by adding three new assessment factors focusing on the quality of exposure assessment (robustness of exposure measurement, variability of exposure intensity or duration, and specificity) and the ability to identify exposure windows (preconception, pregnancy, childhood). Because this tool was developed mainly for randomized clinical trials, we developed ad hoc guidelines to apply the 15 quality rating factors to observational studies [see Supplemental Material, Appendix 3 (doi:10.1289/ehp.0900582.S1)]. D.T.W. and M.C.T. independently scored the studies without blinding to authorship or publication status of the original studies and resolved any scoring differences by consensus. The maximum total possible quality score was 20; the assigned scores ranged from 4 to 17, with a median of 12. Median scores for total quality and its components were based on all studies.

### Analysis

We conducted meta-analyses using the software package Comprehensive Meta Analysis version 2 (Biostat, Inc. 2007). Random-effects summary ORs and 95% CIs were estimated to provide an indicator of the overall strength of associations between pesticide exposure indices and childhood leukemia. We assessed heterogeneity across individual studies using Cochran’s Q-test. Subgroup analyses assessed summary ORs stratified by total quality score, the four quality-score components (external validity, control of bias, exposure assessment, and control of confounding), exposure time window definition (well- or ill-defined preconceptual paternal or prenatal maternal exposure), timing of report of occupational exposure (exposure reported before vs. after offspring leukemia diagnosis), place of occupational exposure (farm, nonfarm, mixed, or unknown), cell type (ALL, AML, or unspecified acute leukemia), type of pesticide (unspecified pesticides, insecticides, herbicides, or fungicides), date of study report (pre-1990, ≥ 1990), and study design (population-based case–control, hospital-based case–control, or cohort). Critical exposure time windows were defined as pregnancy for mothers and up to 2 years before conception for fathers. Some studies of paternal pesticide exposure only assessed exposure during pregnancy or paternal occupation at birth; we deemed these to be reasonable proxies for preconceptual exposure, assuming that paternal occupations likely did not change from preconception to pregnancy (23 of the 27 paternal occupations were in farming). Analysis of the quality component for bias control was limited to paternal exposure because no studies of maternal exposure had median or higher scores. Publication bias was assessed on the assumption that smaller studies are more likely to be published if they suggest elevated risks. We used Begg and Mazumdar’s test based on the rank correlation (as gauged by Kendall’s tau statistic) between standardized effect sizes and their variances to assess this potential source of bias (Begg and Mazumdar 1994). Asymmetry caused by publication bias is expected to produce higher standard errors for smaller studies with larger effects (producing a larger Kendall’s tau *Z*-score).

## Results

### Study identification

The results of the search strategy and study selection process are detailed in Figure 1. From a total of 1,775 studies identified, 111 were retained from the primary screening of abstracts; most excluded studies were irrelevant (*n* = 1,178), duplicates (*n* = 380), or review articles (*n* = 93). After the secondary screening of full reports, a total of 35 studies were deemed eligible.

### Study characteristics

We included 31 of the 35 eligible studies in the meta-analyses: 26 case–control studies and 5 cohort studies [see citations in Table 1 and study summaries in Supplemental Material, Appendix 2 (doi:10.1289/ehp.0900582.S1)]. Among the four excluded studies, three did not present ORs and CIs or sufficient data to enable their calculation (nor were we able to obtain these from the corresponding author) (Buckley et al. 1994; Gold et al. 1982; Hemminki et al. 1981). The other excluded study reported an exceptionally strong association between childhood ALL and pesticide exposure (crude OR = 126.4; 95% CI, 22.2–2,657; calculated from data in the report and assuming one exposed control father rather than none as reported) (Zorlu et al. 2002). This study did not distinguish occupational versus residential pesticide exposure.

Four of the included studies had data for maternal exposure only, and 16 studies had data for paternal exposure only. We conducted meta-analyses of 27 studies with any paternal occupational pesticide exposure with a total of 30 ORs because three studies reported data separately for ALL and AML. For any prenatal maternal occupational pesticide exposure, we analyzed 14 studies with a total of 16 ORs because two studies reported data separately for ALL and AML.

Parental occupational pesticide exposure indices reported by individual studies are shown in Table 1 [see also Supplemental Material, Appendix 4 (doi:10.1289/ehp. 0900582.S1)]. Paternal occupational pesticide exposure information was collected from fathers or proxies after offspring were diagnosed with leukemia in 18 case–control studies, and before offspring leukemia diagnosis in 9 studies, the sources being paternal occupation on birth records in four case–control studies and census, employer, or pesticide applicator records in five cohort studies. Preconceptual paternal occupational pesticide exposure was well defined or reasonably inferable in 15 studies and ill defined in the remaining 12 studies [Table 1; see also Supplemental Material, Appendix 4 (doi:10.1289/ehp.0900582.S1)]. Paternal occupational exposure to unspecified pesticides was usually based on employment in farming or job titles where pesticide exposure commonly occurs. Exposure to specific or broad classes of pesticides was limited to five studies with relevant data collection and results (Danila 1989; Heacock et al. 2000; Infante-Rivard and Sinnett 1999; Monge et al. 2007; Wen et al. 2000).

Maternal occupational pesticide exposure during pregnancy was well defined in 15 studies and ill defined in 1 study.

### Quality assessment

The quality factor scores for included studies are in the Supplemental Material [Appendix 5 (doi: 10.1289/ehp.0900582.S1)]. Compared with lower ranking studies, those with median or higher total quality scores tended to have higher scores for factors related to exposure measurement and bias control (Appendix 5). All five cohort studies had median or higher quality scores.

### Publication bias

We attempted to identify all relevant original studies, including thesis dissertations, in any language (one report was translated from Japanese) (Kishi et al. 1993). Inverse funnel plots of the main findings from studies of any paternal and maternal pesticide exposure and childhood leukemia risk revealed no clear evidence of publication bias; Kendall’s tau *Z*-scores and one-tailed *p*-values for paternal and maternal exposure, respectively, were 0.45, *p* = 0.33, and 0.18, *p*= 0.43 (Table 2).

### Data synthesis

#### Paternal occupational pesticide exposure

**Figure 2:**
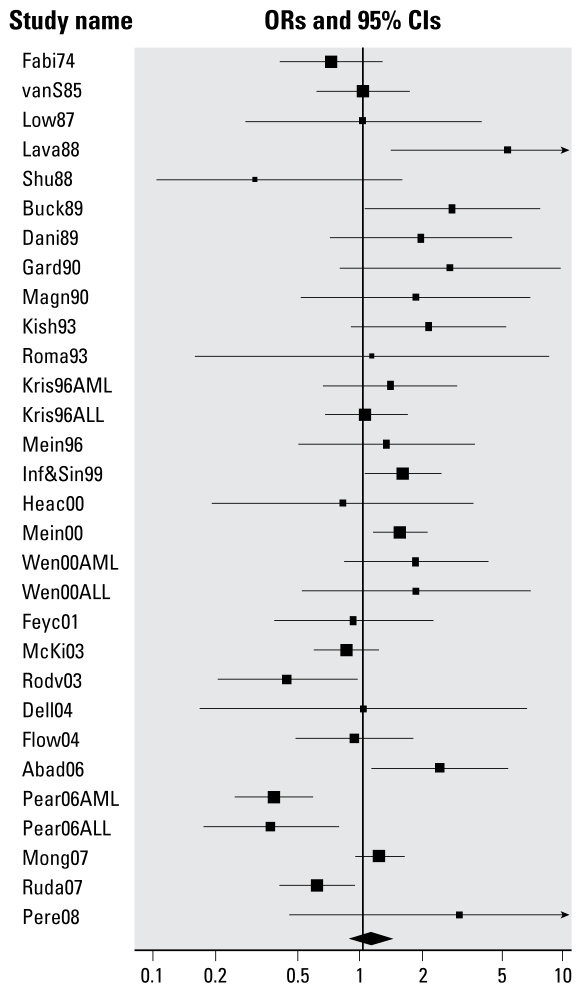
Random effect ORs for childhood leukemia in relation to paternal occupational exposure to any or unspecified pesticides. See Table 1 for list of studies. Some studies reported data separately for AML and ALL.

Results for the 27 studies of any paternal occupational pesticide exposure are shown in Figure 2, arrayed by year of publication; 30 ORs are shown because three studies reported data for ALL and AML separately. Childhood leukemia was not associated with paternal occupational exposure to any pesticides (i.e., exposure to specified or unspecified types of pesticides) (random effects summary OR = 1.09; 95% CI, 0.88–1.34) or unspecified pesticides (random effects summary OR = 1.04; 95% CI, 0.83–1.31) (Table 2). For both analyses, there was evidence of heterogeneity. There was a weak inverse association of borderline statistical significance between the year of publication and ORs of individual studies [e.g., for any paternal occupational pesticide exposure, meta-regression slope = −0.012 (weighted average change in OR per year), *p* = 0.09].

There was an association of borderline statistical significance between childhood leukemia and any paternal occupational pesticide exposure among studies with below-median total quality scores (summary OR = 1.39; 95% CI, 0.99–1.95) but not in those with higher scores (summary OR = 0.93; 95% CI, 0.71–1.21) (Table 3). In analyses of the four quality-score components (external validity, control of bias, exposure measurement, and control of confounding), there was an inverse association between childhood leukemia and any paternal occupational pesticide exposure among studies with median or higher bias control scores (summary OR = 0.73; 95% CI, 0.53–0.99) and a positive association in studies with median or higher pesticide exposure measurement scores (summary OR = 1.37; 95% CI, 1.00–1.89). Childhood leukemia was associated with any paternal occupational pesticide exposure among studies with ill-defined preconceptual exposure windows (summary OR = 1.36; 95% CI, 1.00–1.85) but not those with well-defined windows (summary OR = 0.89; 95% CI, 0.67–1.19).

There was no association between any paternal occupational pesticide exposure and unspecified acute leukemia (summary OR = 0.99; 95% CI, 0.74–1.33) or AML (summary OR = 1.12; 95% CI, 0.60–2.13); the summary OR for ALL was elevated but was not statistically significant (summary OR = 1.30; 95% CI, 0.86–1.94). Childhood leukemia and any paternal occupational pesticide exposure were associated in studies in which exposure information was collected after diagnosis of offspring leukemia (summary OR = 1.34; 95% CI, 1.05–1.70) but not when pesticide exposure information was collected before off-spring leukemia diagnosis (OR = 0.73; 95% CI, 0.54–1.00). Childhood leukemia risk was not elevated in studies of paternal farm-related pesticide exposure (summary OR = 1.04; 95% CI, 0.82–1.32) and was statistically nonsignificantly elevated in studies of nonfarm workplace exposure (summary OR = 1.41; 95% CI, 0.66–3.00) and mixed or unknown workplace exposure (summary OR = 1.30; 95% CI, 0.65–2.60). Childhood leukemia was not associated with any paternal occupational pesticide exposure in pre-1990 or more recent studies [summary ORs, 1.23 (95% CI, 0.76–2.00) and 1.06 (95% CI, 0.83–1.35), respectively] in population-based or hospital-based case–control studies [summary ORs, 1.17 (95% CI, 0.87–1.58) and 1.11 (95% CI, 0.72–1.69), respectively], or in cohort studies (summary OR = 0.88; 95% CI, 0.55–1.40) (Table 3). There were elevated childhood leukemia risks for paternal occupational exposure to the broad pesticide classes of insecticides (summary OR = 1.43; 95% CI, 1.06–1.92), herbicides (summary OR = 1.25; 95% CI, 0.94–1.66), and fungicides (summary OR = 1.66; 95% CI, 0.87–3.17) (Table 3).

#### Prenatal maternal occupational pesticide exposure

**Figure 3:**
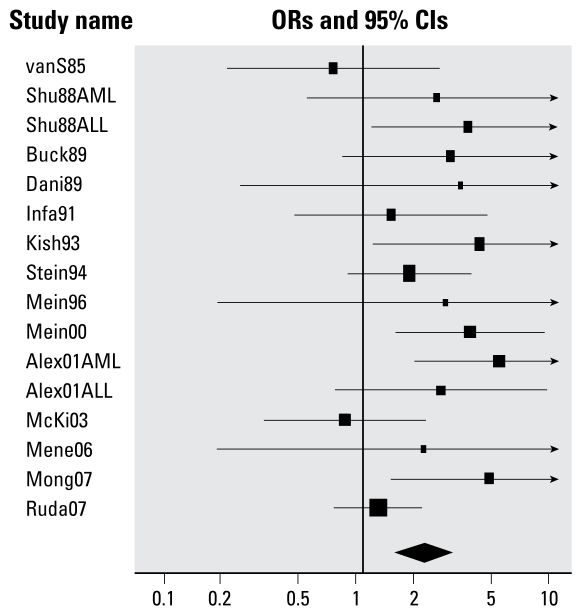
Random effect ORs for childhood leukemia in relation to maternal occupational exposure to any or unspecified pesticides. See Table 1 for list of studies. Some studies reported data separately for AML and ALL.

Childhood leukemia was associated with prenatal maternal occupational exposure to any pesticides (summary OR = 2.09; 95% CI, 1.51–2.88) and unspecified pesticides (summary OR = 2.16; 95% CI, 1.51–3.08), with no evidence of significant heterogeneity (e.g., for any pesticide exposure, *Q* = 19.6, *p* = 0.19) (Table 2). There was no association between year of publication and ORs of individual studies (regression slope = −0.013, *p* = 0.48). Results for each of the studies are shown in Figure 3, sorted by year of publication. The strength of the association was somewhat weaker among studies with median or higher total quality scores (summary OR = 1.86; 95% CI, 1.11–3.14) and those with high confounding scores (summary OR = 2.38; 95% CI, 1.56–3.62) compared with those with lower scores (Table 4). In analyses of quality-score components, the summary ORs in studies with below-median scores for external validity and confounding control were similar to those for studies with higher scores (Table 4). The association was stronger in studies with median or higher exposure measurement scores (summary OR = 2.45; 95% CI, 1.68–3.58) compared with those with lower scores (summary OR = 1.44; 95% CI, 0.83–2.51). All studies of prenatal maternal occupational pesticide exposure had below-median bias control scores.

After excluding one study with an ill-defined prenatal exposure window, the association between maternal exposure to any pesticides during pregnancy and childhood leukemia was little changed (summary OR = 2.06; 95% CI, 1.47–2.90). The association was somewhat stronger for both ALL (summary OR = 2.64; 95% CI, 1.40–5.00) and AML (summary OR = 2.64; 95% CI, 1.48–4.71), compared with unspecified acute leukemia (summary OR = 1.59; 95% CI, 1.02–2.47). The association between childhood leukemia and any prenatal maternal occupational pesticide exposure was somewhat stronger in studies of farm-related exposures (summary OR = 2.44; 95% CI, 1.53–3.89) compared with studies of mixed or unknown pesticide exposure place (summary OR = 1.81; 95% CI, 1.17–2.81). Summary ORs were similar for pre-1990 compared with more recently reported studies of prenatal maternal occupational pesticide exposure and childhood leukemia (Table 4). On removal of the only hospital-based case–control study, the summary OR for any prenatal maternal occupational pesticide exposure was virtually unchanged. All of the studies of prenatal maternal occupational pesticide exposure collected exposure information after offspring leukemia diagnosis. Childhood leukemia was also associated with prenatal maternal occupational exposure to the broad pesticide classes of insecticides (summary OR = 2.72; 95% CI, 1.47–5.04) and herbicides (summary OR = 3.62; 95% CI, 1.28–10.3), but these estimates are based on few studies (Table 4).

## Discussion

After a systematic retrieval and screening of the literature on the relationships between parental occupational pesticide exposure and childhood leukemia, we evaluated the overall evidence using a quantitative meta-analytic approach. Childhood leukemia was not associated with any paternal occupational pesticide exposure in our analyses of all relevant studies or in subgroups of studies with median or higher total quality or bias control scores, well-defined or reasonably inferable preconceptual exposure windows, exposure information collected before offspring leukemia diagnosis, farm-related exposure, data for the major leukemia subtypes, population-based case–control or cohort design, or a publication date of 1990 or later. Childhood leukemia was associated with paternal occupational exposure to insecticides and herbicides, but none of the few relevant studies assessed exposure–risk gradients.

Childhood leukemia was associated with prenatal maternal occupational pesticide exposure with no evidence of statistically significant heterogeneity or publication bias. The association was somewhat stronger among studies with higher exposure measurement quality scores and those with farm-related pesticide exposure. Summary ORs were similar for studies of ALL and AML and for pre-1990 or more recent studies. There were moderately strong associations between childhood leukemia and prenatal maternal occupational exposure to insecticides or herbicides based on the few available studies. All of the eligible studies of prenatal maternal occupational pesticide exposure were based on information collected after offspring leukemia diagnosis. There were too few relevant studies for meaningful analyses of maternal occupational exposure to fungicides or for exposure of either parent to individual pesticides.

Interpretation of our meta-analyses is constrained by limitations in the original studies, particularly exposure assessment and potential sources of bias. We attempted to address these issues by conducting a comprehensive literature search (to reduce publication bias) and independent data extraction and study quality assessment by two persons. We also conducted meta-analyses stratified by parent exposed, study quality scores (total and major components), exposure window definition, leukemia subtype, exposure index, farm versus other workplace exposure, study design, publication period, and broad pesticide class. We assessed study quality with a modified Downs and Black tool (Downs and Black 1998) [see Supplemental Material, Appendix 3 (doi:10.1289/ehp.0900582.S1)]. The main limitations incurred during quality assessment were incomplete descriptions of study methods and findings in reports of original research, lack of a direct method to assess recall bias, and the largely unknown etiology of childhood leukemia, reducing our ability to assess the control of potential confounders. Studies with median or greater quality scores generally had better exposure assessment and control of potential sources of bias compared with lower ranking studies. Few eligible studies collected exposure information for specific or toxicologically related pesticides. Only three studies collected and assessed exposure frequency or intensity information, and little is known about the etiology of childhood leukemia, apart from ionizing radiation. Accordingly, our results should be interpreted cautiously.

Potential sources of bias in observational epidemiologic studies are well described elsewhere (Rothman and Greenland 1998). Although case–control studies dependent on parental recall of potentially hazardous exposures may be subject to recall bias, nondifferential misclassification of exposure status may be a bigger problem (Infante-Rivard and Jacques 2000). The latter source of bias tends to reduce the chance of detecting a true association between a potential causal factor and an adverse health outcome. In a large case–control study, pesticide exposure was the only self-reported paternal occupational exposure associated with childhood AML; this association persisted when paternal occupational pesticide exposure was inferred from a job–exposure matrix (Buckley et al. 1989). Such findings argue against a major bias arising from self-reported occupational pesticide exposure information. In controlled biomoni-toring field studies of farmers, self-reported pesticide exposure information was a fairly good predictor of body burden, if subjects noncompliant for urine collection or reporting incomplete or inconsistent pesticide use information were removed from analysis (Scher et al. 2008). However, other controlled field studies of farm children and farmers revealed poor correlations between biomonitoring and self-reported pesticide exposure data (Arbuckle et al. 2004; Perry et al. 2006).

Previous reviewers concluded that there were fairly consistent associations between childhood leukemia and parental occupational or residential pesticide exposure (Daniels et al. 1997; Zahm and Ward 1998). Recent reviewers noted that associations were strongest for parental pesticide exposure before and during pregnancy and for childhood exposure to household insecticides (Buffler et al. 2005; Infante-Rivard and Weichenthal 2007) and that prenatal maternal pesticide exposure may be more important than paternal exposure (Brown 2006). A recent meta-analysis of seven case–control studies of adult leukemia and occupational pesticide exposure published during 1990–2005 showed a summary OR of 1.35 (95% CI, 0.91–2.0) (Merhi et al. 2007). A meta-analysis of 17 studies of adult myeloid leukemia and occupational pesticide exposure published during 1979–2005 revealed a slightly elevated risk (summary OR = 1.21; 95% CI, 0.99–1.48) (Van Maele-Fabry et al. 2007); their subgroup analyses showed stronger associations in the five studies of pesticide applicators (summary OR = 2.14; 95% CI, 1.39–3.31) and the two studies of manufacturing workers (summary OR = 6.32; 95% CI, 1.90–21.0). Although these studies suggest a role for pesticides in adult leukemia, their relevance to childhood leukemia is not clear because the mechanisms may differ.

Childhood leukemia is associated with genetic polymorphisms in genes encoding enzymes or other proteins involved in DNA repair, membrane transport, cell cycle regulation, and phase I and II metabolism of chemical toxicants (Kim et al. 2006). As noted in a recent review (Anderson 2008), associations between childhood hematopoietic cancers and genetic polymorphisms in genes encoding phase I and II enzymes are consistent with potential chemical causes of these cancers. For instance, a large Quebec case-only analysis reported relatively large interaction ORs between childhood leukemia and *CYP1A1m1* and *CYP1a1m2* variants and prenatal maternal or childhood pesticide exposure (Infante-Rivard et al. 1999).

Most childhood leukemia cases have gross chromosomal abnormalities, including translocations caused by faulty repair of double-strand DNA breaks. Double-stranded DNA breaks may be caused directly by ionizing radiation and certain mutagenic chemicals or indirectly by modulation of type II topoisomerase enzymes. Analysis of routinely collected neonatal blood samples revealed leukemia clones with specific chromosomal translocations in children who later developed ALL, suggesting that many such cases originate *in utero* (Gale et al. 1997). About half of all childhood leukemia cases occur by 3 years of age, and most cases probably have a clonal origin, developing from a single abnormal precursor cell over a period of several months (Ford et al. 1998; Ma et al. 1999; Mori et al. 2002; Taub and Ge 2004). In a small study of infants born in an agricultural region with high pesticide use in the Philippines, the prevalence of the t(8;21) translocation in cord blood samples was 20.5% among those with detectable meconium levels of the methylcarbamate insecticide propoxur, compared with 10% among infants with undetectable levels (crude OR = 2.32; 95% CI, 0.30–57.4; calculated from data given in the report) (Lafiura et al. 2007). It appears that preleukemic clones can persist during childhood and that only a minority progress to leukemia, suggesting that postnatal exposures could influence progression (Maia et al. 2004).

The biological plausibility of potential causal relationships between cancer and pesticide exposure is supported by reviews of available evidence, mainly from animal studies. The U.S. Environmental Protection Agency (EPA) and other national and international bodies have identified about 165 pesticidal active ingredients as known, probable, or possible human carcinogens, some of which have been banned or restricted (Goldman 1998). The 15 most intensely used pesticides in the United States during 2001, based on the amount of active ingredient sold (U.S. EPA 2004), include three probable human carcinogens (alachlor, metam sodium, and chlorothalonil) and five possible human carcinogens (acetochlor, malathion, metolachlor, pendimethalin, and trifluralin) (U.S. EPA 2007). Among 60 pesticides still used in Canada but banned in one or more Organisation for Economic Co-operation and Development member countries because of health and environmental concerns (David Suzuki Foundation 2006), the insecticides carbaryl and propoxur and the fungicides captan, mancozeb, maneb, and metiram are recognized as probable human carcinogens (U.S. EPA 2007).

In experimental animals, exposure of pregnant females to carcinogens can produce cancer in offspring (Autrup 1993). Lymphomas in mice were induced by transplacental exposure to the fungicides carbendazim or dodecylquanidine acetate together with sodium nitrite (Borzsonyi et al. 1976, 1978). Among men, lymphocyte or sperm DNA damage detectable using the comet assay has been associated with background exposure to chlorpyrifos or carbaryl (Meeker et al. 2004), with occupational exposure to carbofuran (Zeljezic et al. 2007) or multiple pesticides (Liu et al. 2006), and with occupations in pesticide production (Bhalli et al. 2006) and farming (Naravaneni and Jamil 2007). Male mice preconceptually exposed to ionizing radiation had increased sperm DNA strand breaks, and their offspring demonstrated an increased risk of hematopoietic cancers (Hoyes et al. 2001). These studies suggest potential mechanisms for relationships between childhood hematopoietic cancers and prenatal maternal or preconceptual paternal pesticide exposures.

## Conclusion

Based on the present meta-analysis of original epidemiologic studies of childhood leukemia and parental occupational pesticide exposure, we concluded that there was no overall association between childhood leukemia and any paternal occupational pesticide exposure among all studies combined or subgroups of studies with high total-quality scores, well-defined or reasonably inferable preconceptual exposure windows, pesticide exposure information collected before offspring leukemia diagnosis, farm-related exposures, or cohort design. We found elevated childhood leukemia risks in relation to paternal occupational exposure to the broad pesticide classes of insecticides and herbicides; however, there were few relevant studies and they did not address exposure–risk relationships, precluding firm conclusions.

We also concluded that there was an overall association between childhood leukemia and prenatal maternal occupational pesticide exposure; this association was somewhat stronger among the subgroups of studies with high exposure-measurement-quality scores or farm-related exposures and those that assessed ALL and AML subtypes. We also found associations between childhood leukemia and maternal occupational exposure to insecticides and herbicides; however, because these were based on few available studies, further research in this area is needed.

Although the evidence for associations between parental occupational pesticide exposure and childhood leukemia is limited, precautionary public health policies that will minimize such exposures may be warranted. The epidemiologic and biological evidence summarized here suggests that avoidance of prenatal maternal occupational pesticide exposure may be particularly important in this regard.

Important research needs include *a*) validated self-reported pesticide exposure indices for both parents, including specific pesticide exposure questions; *b*) biomonitoring of pesticide levels in occupationally exposed men and women; *c*) continued follow-up of existing well-designed cohort studies, such as the Agricultural Health Study in the United States; *d*) follow-up studies of the children of parents in such cohorts; *e*) new case–control and cohort studies with sufficient statistical power to assess childhood leukemia subtypes, leukemia before 5 years of age, potential precursors of childhood leukemia, exposure–risk gradients, specific or toxicologically related groups of pesticides, and genetic susceptibility markers (including preservation of DNA samples from parents and children to permit future analyses of genetic markers); and *f*) basic research on potential biomarkers of pesticide exposure and mechanisms of childhood leukemia initiation and progression.

## Correction

Many of the values (e.g., ORs, 95% CIs, *p*-values) were slightly different in the manuscript originally published online; they have been corrected here.

## Figures and Tables

**Table 1:** Pesticide exposure by parent, exposure definition, source, and exposure window rating.

Reference	Parent	Pesticide exposure definition	Exposure source	Exposure rating^a^
Fabia and Thuy 1974 (Fabi74)	P	Occupation in farming	Birth records	1
van Steensel-Moll et al. 1985 (vanS85)	P	Occupational pesticide exposure during pregnancy	Self^b^	1
	M	Same	Self	1
Lowengart et al. 1987 (Low87)	P	Occupation in farming 1 year before conception to 1 year before diagnosis	Self	2
Shu et al. 1988 (Shu88)	P	Occupation in farming during pregnancy	Self	1
	P	Same	Self	1
Laval and Tuyns 1988 (Lava88)	P^c^	Occupational pesticide exposure, timing not stated	Self	2
Buckley et al. 1989 (Buck89)	P	Occupational pesticide exposure 1 year before birth to diagnosis	Self	2
	M	Same	Self	2
Danila 1989 (Dani89)	P	Agricultural pesticide use since 16 years of age	Self	2
	M	Prenatal agricultural pesticide use	Self	1
Gardner et al. 1990 (Gard90)	P	Occupation in farming	Birth records	1
Magnani et al. 1990 (Magn90)	P	Occupation in farming before child’s birth	Self	2
Infante-Rivard et al. 1991 (Infa91)	M	Occupational pesticide exposure during pregnancy	Self	1
Kishi et al. 1993 (Kish93)	P	Occupational pesticide exposure during pregnancy	Self	1
	M	Same	Self	1
Roman et al. 1993 (Roma93)	P	Occupation in farming	Birth records	1
Steinbuch 1994 (Stein94)	M	Occupational pesticide exposure during pregnancy	Self	1
Kristensen et al. 1996 (Kris96)	P	Occupation as farmer and information on pesticide purchases	Census	2
Meinert et al. 1996 (Mein96)	P	Occupational pesticide exposure during year before conception	Self	1
	M	Occupational pesticide exposure during pregnancy	Self	1
Infante-Rivard and Sinnett 1999 (Inf&Sin99)	P	Preconceptual occupational pesticide exposure, duration not given	Self	2
Heacock et al. 2000 (Heac00)	P	Cumulative chlorophenate exposure hours	Employee records	2
Meinert et al. 2000 (Mein00)	P	Occupational pesticide exposure during year before conception	Self	1
	M	Occupational pesticide exposure during pregnancy	Self	1
Wen et al. 2000 (Wen00)	P	Occupational herbicide exposure up to ≥ 15 years before conception	Self	2
Feychting et al. 2001 (Feyc01)	P	Job title with likely pesticide exposure 2–26 months before child’s birth	Census	1
Alexander et al. 2001 (Alex01)	M	Occupational pesticide exposure during pregnancy	Self	1
McKinney et al. 2003 (McKi03)	P	Agricultural chemical use during 1 year before child’s birth	Self	1
	M	Same	Self	1
Rodvall et al. 2003 (Rodv03)	P	Pesticide applicator up to 29 years before child’s birth	License	2
Dell 2004 (Dell04)	P	Occupational pesticide exposure during 2 years before conception	Self	1
Flower et al. 2004 (Flow04)	P	Farm pesticide applicator during wide preconceptual period	License	2
Abadi-Korek et al. 2006 (Abad06)	P	Occupational pesticide exposure before date of diagnosis	Self	2
Menegaux et al. 2006 (Mene06)	M	Occupational pesticide exposure during pregnancy	Self	1
Pearce et al. 2006 (Pear06)	P	Occupation in farming	Birth records	1
Monge et al. 2007 (Mong07)	P	Occupational pesticide exposure during year before conception	Self	1
	M	Occupational pesticide exposure during pregnancy	Self	1
Rudant et al. 2007 (Ruda07)	P	Occupation in farming during pregnancy	Self	1
	M	Occupational pesticide exposure during pregnancy	Self	1
Perez-Saldivar et al. 2008 (Pere08)	P	Occupational pesticide exposure during 2 years before conception	Self	1

Abbreviations: M, maternal; P, paternal.

a Ratings: 1, reported to be exposed during 2 years before conception (for fathers) or pregnancy (for mothers) or such exposure was reasonably inferable; 2, ill-defined exposure time window.

b Reported by given parent or spouse.

c Study reported paternal or maternal occupational pesticide exposure, assumed here to be mainly paternal.

**Table 2:** Random effects summary ORs for childhood leukemia in relation to parental occupational pesticide exposure.

Exposure (no. of risk estimates)^a^	Summary OR (95% CI)	Heterogeneity *Q*-value	Publication bias (Kendall’s tau *Z*-score)	Meta-regression slope^b^
Paternal occupational exposure				
Any pesticide exposure^c^ (*n* = 30)	1.09 (0.88–1.34)	81.0, *p* < 0.001	0.45, *p* = 0.33	−0.012, *p* = 0.09
Unspecified pesticides only^d^ (*n* = 26)	1.04 (0.83–1.31)	76.9, *p* < 0.001	0.51, *p* = 0.31	−0.011, *p* = 0.13
Prenatal maternal occupational exposure				
Any pesticide exposure (*n* = 16)	2.09 (1.51–2.88)	19.6, *p* = 0.19	0.18, *p* = 0.43	−0.013, *p* = 0.48
Unspecified pesticides only (*n* = 14)	2.16 (1.51–3.08)	19.2, *p* = 0.12	0.05, *p* = 0.48	−0.016, *p* = 0.41

a Number of ORs summarized (one per study unless a study reported data separately for ALL and for AML).

b Regression of OR versus calendar year: weighted average change in OR per year.

c Exposed to specified or unspecified types of pesticides.

d Excludes studies that reported only exposure to specific types of pesticides.

**Table 3:** Random effects summary ORs for childhood leukemia in relation to paternal occupational pesticide exposure: subgroup analyses.

Exposure (no. of risk estimates)^a^	Summary OR (95% CI)	Heterogeneity *Q*-value
Total quality score < median (14)	1.39 (0.99–1.95)	19.3, *p* = 0.11
Total quality score ≥ median (16)	0.93 (0.71–1.21)	53.4, *p* < 0.001
External validity score < median (12)	1.06 (0.75–1.51)	42.0, *p* < 0.001
External validity score ≥ median (18)	1.10 (0.84–1.43)	33.7, *p* = 0.009
Bias score < median (20)	1.33 (1.05–1.69)	35.6, *p* = 0.012
Bias score ≥ median (10)	0.73 (0.53–0.99)	23.8, *p* = 0.005
Exposure measurement score < median (19)	0.92 (0.71–1.19)	46.5, *p* < 0.001
Exposure measurement score ≥ median (11)	1.36 (1.00–1.89)	20.0, *p* = 0.03
Confounding score < median (14)	1.17 (0.84–1.63)	24.7, *p* = 0.03
Confounding score ≥ median (16)	1.03 (0.77–1.38)	54.9, *p* < 0.001
Ill-defined exposure window (12)	1.37 (1.00–1.85)	21.1, *p* = 0.10
Well-defined exposure window^b^ (15)	0.89 (0.67–1.19)	52.1, *p* < 0.001
Unspecified acute leukemia (18)	0.99 (0.74–1.33)	32.4, *p* = 0.01
ALL (8)	1.30 (0.86–1.94)	36.0, *p* < 0.001
AML (4)	1.12 (0.60–2.13)	12.5, *p* = 0.006
Exposure reported after diagnosis (19)	1.34 (1.05–1.70)	35.6, *p* = 0.008
Exposure reported before diagnosis (11)	0.73 (0.54–1.00)	24.1, *p* = 0.007
Exposure in farming (23)	1.04 (0.82–1.32)	73.6, *p* < 0.001
Nonfarm exposure (4)	1.41 (0.66–3.00)	1.2, *p* = 0.76
Mixed or unknown exposure place (3)	1.30 (0.65–2.67)	4.5, *p* = 0.11
Pre-1990 (7)	1.23 (0.76–2.00)	13.6, *p* = 0.04
≥ 1990 (23)	1.06 (0.83–1.35)	67.3, *p* < 0.001
Population-based case–control studies (14)	1.17 (0.87–1.58)	30.7, *p* = 0.004
Hospital-based case–control studies (10)	1.11 (0.72–1.69)	49.7, *p* < 0.001
Cohort studies (6)	0.88 (0.55–1.40)	4.9, *p* = 0.43
Insecticides (3)	1.43 (1.06–1.92)	0.33, *p* = 0.85
Herbicides (5)	1.25 (0.94–1.66)	1.9, *p* = 0.75
Fungicides (4)	1.66 (0.87–3.17)	4.64, *p* = 0.20

a Number of ORs summarized (one per study unless a study reported data for ALL and AML separately).

b Or reasonably inferable.

**Table 4:** Random effects summary ORs for childhood leukemia in relation to prenatal maternal occupational exposure: subgroup analyses.

Exposure (no. of risk estimates)	Summary OR (95% CI)	Heterogeneity *Q*-value
Total quality score < median (12)	2.25 (1.49–3.42)	13.3, *p* = 0.27
Total quality score ≥ median (4)	1.86 (1.11–3.14)	5.0, *p* = 0.17
External validity score < median (7)	1.99 (1.12–3.51)	10.7, *p* = 0.10
External validity score ≥ median (9)	2.18 (1.43–3.31)	9.0, *p* = 0.34
Exposure measurement score < median (7)	1.44 (0.83–2.51)	5.6, *p* = 0.47
Exposure measurement score ≥ median (9)	2.45 (1.68–3.58)	11.9, *p* = 0.16
Confounding score < median (7)	1.71 (0.99–2.96)	8.9, *p* = 0.18
Confounding score ≥ median (9)	2.38 (1.56–3.62)	10.3, *p* = 0.24
Well-defined^a^ exposure window (15)	2.06 (1.47–2.90)	19.2, *p* = 0.16
Unspecified acute leukemia (7)	1.59 (1.02–2.47)	11.0, *p* = 0.09
ALL (5)	2.64 (1.40–5.00)	1.8, *p* = 0.77
AML (4)	2.64 (1.48–4.71)	2.8, *p* = 0.42
Exposed on farm (9)	2.44 (1.53–3.89)	8.7, *p* = 0.37
Mixed or unknown exposure place (7)	1.81 (1.17–2.81)	9.2, *p* = 0.16
Pre-1990 (5)	2.12 (1.05–4.25)	4.0, *p* = 0.40
≥ 1990 (11)	2.10 (1.44–3.08)	15.5, *p* = 0.11
Population-based case–control studies (15)	2.10 (1.50–2.94)	19.6, *p* = 0.14
Insecticides (6)	2.72 (1.47–5.04)	6.2, *p* = 0.29
Herbicides (2)	3.62 (1.28–10.3)	0.8, *p* = 0.37

a Or reasonably inferable.
